# The Bluetooth Mesh Standard: An Overview and Experimental Evaluation

**DOI:** 10.3390/s18082409

**Published:** 2018-07-25

**Authors:** Mathias Baert, Jen Rossey, Adnan Shahid, Jeroen Hoebeke

**Affiliations:** Department of Information Technology, Ghent University/imec, Technologiepark-Zwijnaarde 15, 9052 Ghent, Belgium; jen.rossey@ugent.be (J.R.); adnan.shahid@ugent.be (A.S.); jeroen.hoebeke@ugent.be (J.H.)

**Keywords:** BLE, Bluetooth Mesh, round-trip-time, IoT, scalability, performance, interoperability

## Abstract

Mesh networks enable a many-to-many relation between nodes, which means that each node in the network can communicate with every other node using multi-hop communication and path diversity. As it enables the fast roll-out of sensor and actuator networks, it is an important aspect within the Internet of Things (IoT). Utilizing Bluetooth Low Energy (BLE) as an underlying technology to implement such mesh networks has gained a lot of interest in recent years. The result was a variety of BLE meshing solutions that were not interoperable because of the lack of a common standard. This has changed recently with the advent of the Bluetooth Mesh standard. However, a detailed overview of how this standard operates, performs and how it tackles other issues concerning BLE mesh networking is missing. Therefore, this paper investigates this new technology thoroughly and evaluates its performance by means of three approaches, namely an experimental evaluation, a statistical approach and a graph-based simulation model, which can be used as the basis for future research. Apart from showing that consistent results are achieved by means of all three approaches, we also identify possible drawbacks and open issues that need to be dealt with.

## 1. Introduction

Bluetooth Low Energy (BLE) first appeared in the Bluetooth 4.0 standard [1], which made a clear difference between Classic Bluetooth and the Low Energy variant (also labeled Bluetooth Smart). Low-power wireless communication scenarios could use this new technology to satisfy the necessities of communication while saving more power. In recent years, BLE has profiled itself as one of the leading technologies for the Internet of Things [2] and has been implemented as a household feature in current smartphones, tablets, laptops, etc. This way, users could immediately interact with these BLE products without having to purchase an additional gateway. With each new version of the technology, the achievable throughput, range and energy efficiency have improved significantly [3].

However, the whole Bluetooth Low Energy design was focused on these phone-device interactions, resulting in typical point-to-point, star-based network topologies, with the phone being the central of the network. This approach did not have the mesh capabilities offered by its low-power competitors such as ZigBee and Thread, both running on top of IEEE 802.15.4 in the 2.4 GHz band. Meshing enables a topology where each node in the network can talk to every other node, directly or via multi-hop communication. Such a network is self-organizing, self-healing and enables path diversity.

Consequently, several attempts were made to add meshing capabilities to BLE, resulting in different techniques, which are discussed in detail in [4]. This survey states that there are still some open issues regarding BLE mesh networking. One of them is the interoperability between the different implementations, an issue that can only be resolved when BLE meshing is standardized. This is exactly what happened recently, when the Bluetooth SIG group released a mesh network specification based on BLE by making use of its advertising capabilities [5].

As such, it is relevant to find out how this new standard operates, tackles the other open issues identified in [4] and handles the disadvantages of using the advertising capability of BLE as a basis for communication. This is exactly the key contribution of this paper. Apart from giving the reader an overview on how the BLE meshing specification operates, insights are given in its performance by means of three approaches: an experimental evaluation, a statistical approach and using a theoretical model.

The rest of the paper is organized as follows. In Section 2 and Section 3, we introduce the reader to Bluetooth Low Energy itself as well as the new Bluetooth Mesh standard. Section 4 briefly describes related work. In Section 5, we first take a statistical approach to calculate the round-trip-time (RTT), which is further used to validate our experiments presented in Section 6. This section also contains an overview of the used hardware and measuring environment. The experiments focus on the RTT as a metric. Section 7 introduces a theoretical model for the Bluetooth Mesh standard. The model is validated through the experiments in the previous section. Finally, we give some concluding remarks and discuss open issues in Section 8.

## 2. Background on Bluetooth Low Energy

**Figure 1:**
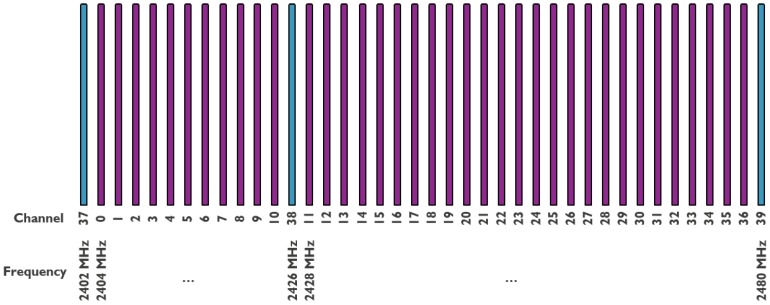
Distribution of channels for BLE communication.

As stated in Section 1, BLE is the low-power variant of Classic Bluetooth. It operates in the same 2.4 GHz ISM band and uses frequencies between 2402 and 2480 MHz. The used spectrum is divided into several channels, but, unlike Classic Bluetooth, BLE uses channels of 2 MHz spacing, which leads to 40 usable channels. This is shown in Figure 1. These 40 channels are divided into three primary advertisement channels (37, 38 and 39), avoiding the main channels used by Wi-Fi, and 37 connection oriented channels. BLE offers two modes of communication between devices: advertising mode or connection oriented mode.

**Figure 2:**
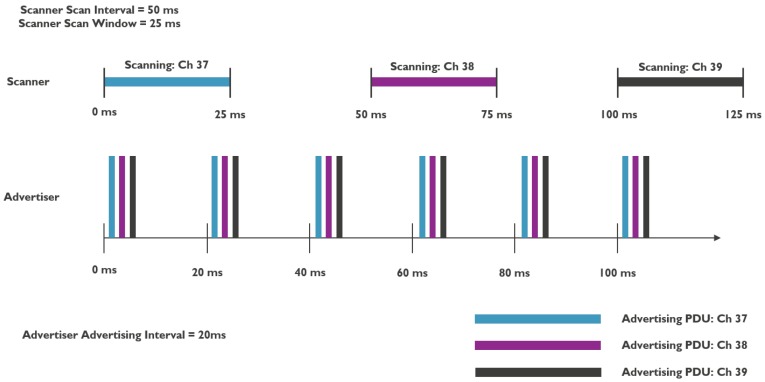
Illustration of BLE advertising and scanning modes [6].

The advertising mode means that one or more devices advertise information on the three advertisement channels at a certain interval (= broadcaster or advertiser role) while other devices in the range of those broadcasters scan on the advertisement channels, one at a time (also at a certain interval and only during a specific window), to possibly pick up that information (= scanner role). This is illustrated in Figure 2.

**Figure 3:**
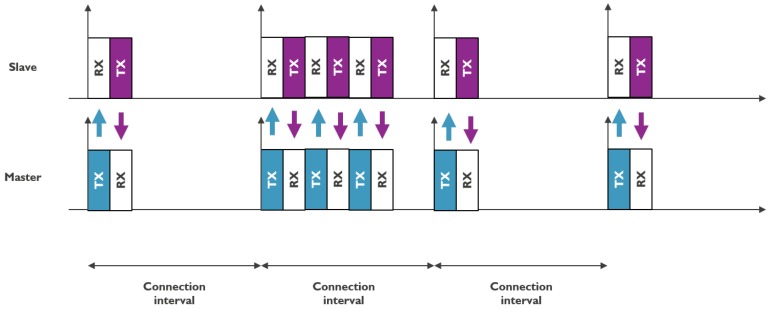
Illustration of BLE connection oriented mode [6].

The connection oriented mode uses the other 37 channels in a more synchronized way. One device fulfills the role of a master (or central) and manages connections to several slaves (or peripherals), as shown in Figure 3. The master manages and synchronizes these connections via a TDMA scheme and each connection uses a FHSS technique to ensure robustness. Information is sent on one of these channels at a certain interval and on a different channel each time.

Both communication techniques have their use and both of them can be used to realize BLE mesh networking. In general, broadcasting implementations use broadcasting and scanning as it is, with some extra logic on top of each node to ensure that received packets are rebroadcasted further into the network. Connection oriented implementations mostly rely on the possibility of a node to be both a central and a peripheral simultaneously, thus linking several star topologies together into a mesh topology. For both approaches, several implementations already exist [4], with each approach having its own advantages and disadvantages. Depending on the use case, one of the two could offer a more satisfying solution. However, the Bluetooth Special Interest Group (SIG) has chosen to use the advertising mode as the core underlying technique for the Bluetooth Mesh Standard, which is discussed in detail in the next section.

## 3. Primer on the Bluetooth Mesh Standard

### 3.1. Bluetooth Mesh Concept

**Figure 4:**
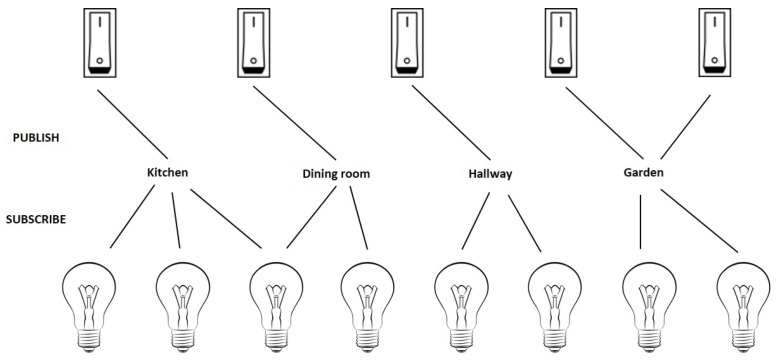
Conceptual definition of the Bluetooth Mesh Standard through a publish/subscribe model [5].

Conceptually, the Bluetooth Mesh Standard is defined as a publish/subscribe model where publishers can publish to a certain topic and subscribers can subscribe to one or more topics of interest. This is illustrated in Figure 4, where switches can publish to a specific topic and lamps can subscribe to one or more topics. This concept is used as an inspiration for the implementation in the standard. A node in a Bluetooth Mesh network can subscribe to one or more addresses (stored in the *subscriber list*) and publish to one specific address (stored in the *publish address*). The standard defines two main types of addresses: a unicast and group address. A unicast address is given to each node when it becomes part of the network and uniquely identifies this node. A group address represents a group of nodes.

Each node has its unicast address in the subscriber list. If the node wants to join a specific group, it has to add the corresponding group address to the subscriber list as well. Another node can send a message to this node using its specific unicast address or using a group address that the node has subscribed to. This information is stored in the publish address of the sending node.

### 3.2. Bluetooth Mesh Topology

To be able to connect these different publishers and subscribers, a mesh topology is created. The remainder of this section gives a step by step overview of all different types of nodes present in such a topology.

The standard uses BLE advertising and scanning as an underlying technology to implement communication. To communicate in a Bluetooth Mesh network, a flooding mechanism is used. By default, a flooding mechanism ensures that each node in the network repeats incoming messages, so that they are relayed further, until the destination node is reached. Compared to normal BLE advertising, Bluetooth Mesh nodes do not send their packets according to an advertising interval. They send their packets directly after a random generated backoff time.

To scan the advertisement channels for incoming packets, the mesh nodes use a 100% duty cycle. This means that nodes in the mesh are always scanning, unless they are sending a packet. For scanning, the scan interval and window are still used. The scan window is equal to the scan interval, to ensure that a node never stops scanning. The scan interval makes sure that a node switches between the advertisement channels to scan.

**Figure 5:**
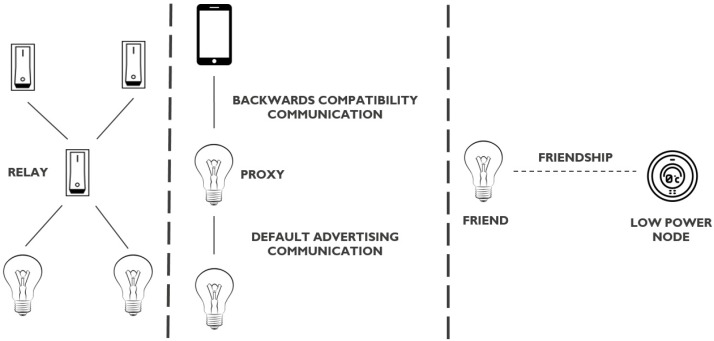
An example of each optional feature within the Bluetooth Mesh standard.

The standard uses a new type of BLE advertisement packet to communicate in a mesh network, which is only supported by devices that support both BLE and Bluetooth Mesh. Fortunately, the standard also defines a backwards compatibility feature to ensure that BLE devices which do not support Bluetooth Mesh can also be part of a Bluetooth Mesh network. This feature is based on BLE connections. Next to this, a Bluetooth Mesh node can implement some optional features, meant to manage and enhance communication, as indicated by Figure 5. The features are explained in the following subsections.

#### 3.2.1. Relay Feature

Without proper management, the flooding mechanism used in the standard would reduce scalability, robustness, etc. severely. To prevent this, the relay feature has been introduced. In short, only nodes that have the relay feature enabled will forward received messages further into the network.

The standard also introduces a message cache that ensures that a relay only relays a specific message once, as well as a Time-To-Live (TTL) field for the messages. A relay node only relays a message if the message is not in the cache and its TTL is greater than 1. It always reduces the TTL by 1 before relaying further into the network.

In Figure 5, this feature is indicated on the left. The switches above can only reach the lamps below, via the switch in between. The intermediate switch is configured to act as a relay.

#### 3.2.2. Proxy Feature

The standard defines a backwards compatibility feature for BLE devices that do not support Bluetooth Mesh. This way, a native BLE device such as a smartphone, can also connect with a Bluetooth Mesh network. This is achieved by means of the proxy feature. A node that enables the proxy feature can communicate in two ways: using the default BLE advertising capability and using the backwards compatibility feature that uses BLE connections.

In Figure 5, this is shown in the middle. The smartphone does not support Bluetooth Mesh. It can still connect with the lamp below through an intermediate lamp that acts as a proxy. The communication between the smartphone and the intermediate proxy lamp is done using the backwards compatibility feature. The intermediate proxy lamp forwards the information further into the network (and thus to the lamp below) using the default BLE advertising feature. This also explains why a proxy node should also be a relay node.

#### 3.2.3. Friendship Feature

The last feature being discussed is the Friendship feature, which consists of two sub features: friend and low-power node. Because of the flooding mechanism that is used in this standard, nodes use a 100% duty cycle to scan the different advertisement channels. This decreases the low energy aspect of BLE advertising and thus limits the usefulness for power sensitive applications.

To cope with this limitation, the Friendship feature has been introduced. A power limited device that wants to become part of the mesh network, is not able to scan at a 100% duty cycle (e.g., because of energy constraints). However, other nodes in the network (e.g., light bulbs that are mains powered) could assist this node so that it can still be part of the network. Both nodes can establish a friendship, with the power limited device acting as a low-power node and the light bulb node acting as a friend node.

A friend node is responsible for two things: storing incoming messages for his low-power node and relaying received messages from his low-power node further into the network. As such, every friend node should also be a relay node. The low-power node asks his friend node for new messages at a certain interval. Using this feature, the low-power node does not need to have a 100% duty cycle and can save power.

On the right in Figure 5, two nodes are shown: a lamp and a sensor. The lamp acts as friend node and the sensor acts as low power node. They form a friendship.

### 3.3. Bluetooth Mesh Stack

**Figure 6:**
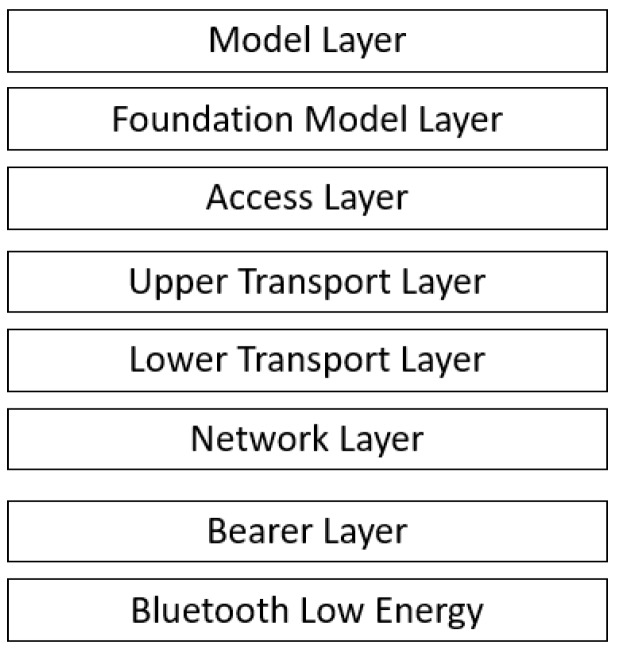
The layered architecture of the Bluetooth Mesh Standard [5].

Each device in a Bluetooth Mesh network implements the Bluetooth Mesh stack shown in Figure 6. This stack is made up as a layered architecture, consisting of the following layers:

- **Bluetooth Low Energy Core Specification:** The standard is built on top of the BLE specification and uses both the advertising and connection oriented mechanisms.
- **Bearer layer:** This layer defines an abstraction for the underlying BLE specification towards the layers above. These abstractions are called *bearers* and denote the bearer of information used to carry information into the network. The standard defines two bearers: ADV bearer and GATT bearer. The ADV bearer abstracts the mechanism that uses BLE advertising and the GATT bearer abstracts the mechanism that uses BLE connections.
- **Network layer:** This layer is responsible for relaying, network level security, etc.
- **Transport layer:** Segmenting bigger messages and reassembling segmented messages is done on this layer. Application level security is handled here as well.
- **Access layer:** Defines the glue between the more application focused layers (application, model and foundation model) and the more technical layers below. It ensures that the correct parts of the application receive incoming messages from the technical layers and that outgoing messages from the application are correctly forwarded to the technical layers below.
- **(Foundation) model layer:** The two highest layers comprise the definition of so called models. A model is a standardization of a specific scenario. This can relate to the configuration and management capabilities of the mesh network (foundation models), as well as to specific user scenarios commonly used (e.g., standardization of a light switch). On these higher layers, a Bluetooth Mesh device can be defined as a combination of both the foundation models and several other models. Each of these models represents a part of the application and together they form a representation of the device as a whole.

On top of this stack, an application is implemented. The standard also defines several compulsory security measures (network and application layer security, key refresh, etc.) and mechanisms for both provisioning and configuring a device to be part of a mesh network. These aspects of the standard are beyond the scope of this paper. In our experiments, we always assumed a fully provisioned and configured mesh network.

### 3.4. Bluetooth Mesh Communication Basics

As the goal of this paper is to evaluate the performance of the Bluetooth Mesh standard in terms of communication latencies, it is important to understand the different steps in the communication flow that influence these latencies.

**Figure 7:**
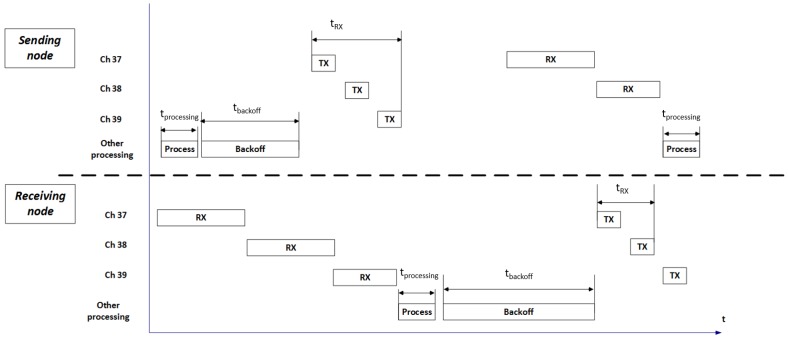
An example of the underlying wireless communication between two nodes in a Bluetooth Mesh network.

Figure 7 shows the communication flow of a reliable communication event between two directly connected mesh nodes. First, an event on the application layer of the sending node (e.g., pressing a button) triggers the need to send a message to another node. It takes some processing time for that message to be sent from top to bottom in the stack. Before the message is sent over the air, a random back-off mechanism is used that holds the message for a random time between 0 ms and $t_{maximum}$ (configurable by the user). Ultimately, the back-off expires and the message is broadcasted. This is done via normal BLE advertising. To recap, a message is advertised on channels 37, 38 and 39 sequentially and a receiving node is scanning on one of these channels, switching from one channel to another channel at a fixed interval. The message is sent on the three advertising channels. The time required to send a message on one channel depends on the size of the packet and the configured bit rate of the BLE radio. The time to switch between channels also needs to be taken into account (disabling the radio, switching the channel and turning the radio back on).

The other node in the communication flow is scanning for packets. Depending on which channel the receiving node is listening during the transmission of the packet, it takes a specific amount of time until the packet is being received. For example, in Figure 7, the receiving node receives the message on channel 39, which means that the full transmit time for the sending node needs to be taken into account.

After receiving the message, the receiving node passes the message up to the application layer, where possibly other events are triggered (e.g., turning on a lamp) after which the node sends an acknowledgement to the sending node. This acknowledgement follows the same pattern as before. However, now the message corresponding to the acknowledgement is received on channel 38, which means that only the transmit time for channels 37 and 38 (as well as the channel switch time) needs to be taken into account. Finally, the sending node processes this acknowledgement, which concludes the communication flow.

## 4. Related Work

Several proprietary and academic solutions for BLE Meshing already exist. The BLE survey in [4] presents a general overview of these solutions and analyzes several approaches to implement a BLE Mesh solution. At that time, the conclusions were relevant to take into consideration when considering how the Bluetooth Mesh standard should evolve.

The work in [7] focuses on a performance evaluation of the two approaches that can be used to implement meshing over BLE. The main conclusion is that an advertising approach leads to a lower end-to-end delay and higher power consumption, compared to a connection oriented approach. The authors propose an architecture that dynamically changes the used approach based on message priority, to be able to maintain a lower power consumption if possible, while ensuring a lower end-to-end delay when needed.

A performance evaluation on Bluetooth Low Energy advertising mode and connection oriented mode is available in [8,9]. A more theoretical study on neighbor discovery in advertising mode is presented in [10] and gives a clear view on the minimum and maximum time between advertising a packet and receiving it through scanning.

However, none of these works have taken into consideration the Bluetooth Mesh standard itself or evaluated its performance. To the best of our knowledge, this is the first work that presents an overview of this new standard along with its performance.

## 5. Statistical Estimation of RTT

To validate the obtained experimental results that are presented in the following sections, we first take a statistical approach to calculate the expected RTT, which is elaborated in this section.

### 5.1. Basic Formula for RTT

Based on the discussion in Section 3.4, the round-trip-time of the exchange can be expressed as:

(1) $$t_{RTT}=\sum_{i=1}^{2}\left(t_{Backoff_{i}}+t_{TX_{i}}\right)+t_{Processing_{total}}$$

This is of course only valid for one-hop communication. In the case of multi-hop communication, the message is relayed by one or more nodes before reaching the end node, resulting in an increased round trip time that can be expressed as:

(2) $$t_{RTT}=\sum_{i=1}^{n}\left(t_{Backoff_{i}}+t_{TX_{i}}\right)+t_{Processing_{total}}$$

Here, n=#hops∗2, assuming both the message and its acknowledgement need the same amount of hops.

Due to several reasons (interference, non-overlapping scanning and advertising channels, etc.) a message (or its acknowledgement) could get lost before it reaches the destination. When reliable messaging is used, the sender may retransmit the message after an arbitrary time period, which is application specific. Considering this, the final round-trip-time for multi-hop communication with potential packet loss is given by

(3) $$t_{RTT}=\sum_{i=1}^{n}\left(t_{Backoff_{i}}+t_{TX_{i}}\right)+t_{Processing_{total}}+\sum_{i=1}^{m}t_{Retransmit_{i}}$$

Here, *m* is the number of failed transmissions and $t_{Retransmit_{i}}$ the time between transmission attempt *i* by the sender and the moment the sender detects the failure.

### 5.2. Average Forwarding Time, All Nodes Listening on Same Advertisement Channel

Of course, when having multiple neighbors, the exact timings when the packets will be relayed by these neighbors will depend on the specific channel each neighboring node is listening on as well as the backoff time it uses until relaying the packet.

To calculate this, we first only look at the backoff time, assuming all nodes are listening on the same channel. Now, consider a network containing a sending node with *n* neighbors. When the sending node broadcasts a packet, ultimately each neighbor receives it (assuming no packet loss). Before these *n* neighbors relay the packet further into the network, each of them independently calculates a backoff time between 0 and $t_{maximum}$. In more statistical terms, these calculations can be seen as a sequence of independent identically distributed (IID) random variables $X_{1},...,X_{n}$. Assuming that each neighbor reaches the same set of nodes with its rebroadcast, it is interesting to know the how fast the first neighbor will rebroadcast the packet. This is represented by the minimum of such a sequence of variables, $minX_{i}=T$. To use this information for the theoretical evaluation, the average of this minimum for a specific amount of *n* variables needs to be found, which is expressed as E[T].

The formula given by D. Seita’s calculations in [11], gives an average of $\frac{1}{n+1}$ for the minimum of *n* IID variables, each of them with a uniform random value in [0,1]. This can be easily extended to variables with a value in [0,$t_{maximum}$] as follows to obtain E[T]. First, a representation for $P(minX_{i}\leq t)$ is revealed.

$$\begin{array}{cc} P(minX_{i}\leq t) & =1-P(minX_{i}\geq t)\\ & =1-P(X_{1}\geq t,...,X_{n}\geq t)\\ & =1-\prod_{i=1}^{n}P\left(X_{i}\geq t\right)\\ & =1-\prod_{i=1}^{n}\left(1-P\left(X_{i}\leq t\right)\right)\\ & =1-{\left(1-P\left(X_{i}\leq t\right)\right)}^{n} \end{array}$$

To find $P(minX_{i}\leq t)$, $P(X_{i}\leq t)$ is needed. As we have *n* IID variables, it does not matter for which $X_{i}$ this value is calculated. Because of the uniformity of the values, $P\left(X_{i}\leq t\right)=\frac{t}{t_{maximum}}$. This way, the density function $f_{T}\left(t\right)$ can be calculated by differentiating $P(minX_{i}\leq t)$, resulting in $f_{T}\left(t\right)=\frac{n}{t_{maximum}}\times {\left(1-\frac{t}{t_{maximum}}\right)}^{n-1}$. Thus, finally, as $E\left[T\right]=\int_{0}^{t_{maximum}}t\times f_{T}\left(t\right)dt$, we obtain that

(4) $$E\left[T\right]=\frac{t_{maximum}}{n+1}$$

### 5.3. Average Forwarding Time, All Nodes Listening on Random Advertisement Channel

However, the backoff time is not the only time that needs to be taken into account for these neighbors. Before a neighboring node can calculate the backoff time, it first needs to receive the complete packet on one of the three advertisement channels. For each neighbor, there is $\frac{1}{3}$ chance that it is listening on one of these three channels at a given moment. Depending on which channel the packet is received on, it will take longer until the node can calculate the next backoff time and relay the packet. Thus, not only the backoff time needs to be taken into account, but also the extra scan time. Let Q denote the extra time needed to receive the packet on channel 38 instead of channel 37. Thus, $t_{37}$ = 0 ms, $t_{38}$ = Q ms and $t_{39}$ = 2Q ms. With this, the worst case total waiting time until a neighbor can relay a packet further becomes $t_{maximum}$ + 2Q ms.

**Figure 8:**
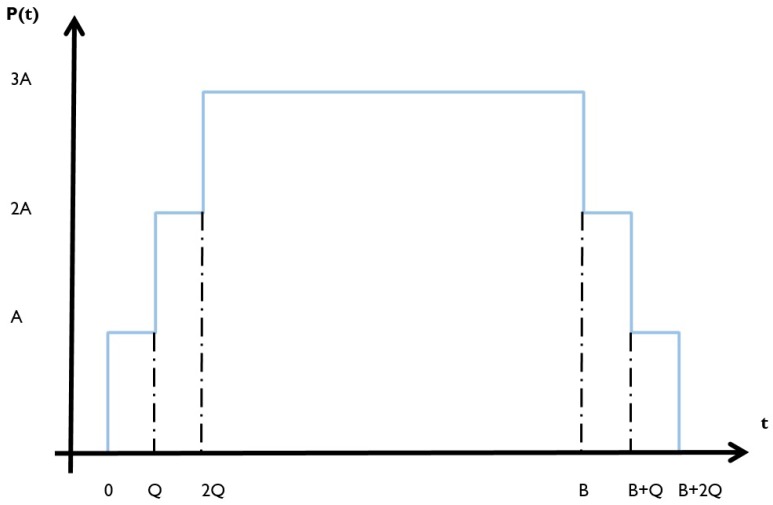
Distribution of the density function for the [0,B+2Q] interval.

The next step is to find a representation for the average minimum of *n* variables, each of them with a value in the range [0,B+2Q] (with B = $t_{maximum}$). However, in these calculations, the chances for each possible value in the defined interval are no longer uniformly distributed. The probability density function f(t) for such a variable is illustrated in Figure 8. Here, *A* represents an unknown chance. The assumption made here is that $Q<\frac{B}{2}$, indicating that the maximum backoff time is larger than twice the channel switching time when broadcasting on the three advertisement channels, which holds in practice.

Nodes that receive a packet on channel 37, have a total delay situated in [0,B]. For channel 38, this is [Q,B + Q] and, for channel 39, this is [2Q,B + 2Q]. Some parts of these intervals overlap. Knowing that each channel has $\frac{1}{3}$ chance of being in use by a particular node, it is clear that some delays have a higher chance of occurring than others, because they can occur in combination with more than one channel. Using this information, it is clear that these chances are related. If there is a chance of having a delay in [0,Q], there is two times more chance of having a delay in [Q,2Q] as it can happen for nodes listening on channel 37 or 38 and three times more chances of having a delay in [2Q,B] as it can happen for nodes listening on channel 37, 38 or 39. Combining this information with the same effect for [B,B + Q] and [B + Q,B + 2Q] explains the distribution depicted in Figure 8.

The value of *A* is still unknown. The chance of being on channel 37 is $\frac{1}{3}$ and the chance of having a specific value in [0,B] when listening on channel 37 is $\frac{1}{B}$. The distribution for P(t) between [0,Q] is uniform and can only occur when receiving on channel 37. Using this information, the value of A is found to be:

$$A=\frac{1}{3}\times \frac{1}{B}=\frac{1}{3B}$$

This means that $2A=\frac{2}{3B}$ and $3A=\frac{1}{B}$. To validate this, we make use of the property that $\int_{-\infty }^{\infty }f\left(t\right)dt=1$.

$$\int_{0}^{B+2Q}f\left(t\right)dt=\int_{0}^{Q}f{\left(t\right)}_{a}dt+\int_{Q}^{2Q}f{\left(t\right)}_{b}dt+\int_{2Q}^{B}f{\left(t\right)}_{c}dt+\int_{B}^{B+Q}f{\left(t\right)}_{d}dt+\int_{B+Q}^{B+2Q}f{\left(t\right)}_{e}dt$$

Filling in the density functions gives:

$$\int_{0}^{B+2Q}f\left(t\right)dt=\frac{Q-0}{3B}+\frac{2\times (2Q-Q)}{3B}+\frac{3\times (B-2Q)}{3B}+\frac{2\times (B-(B+Q))}{3B}+\frac{B+Q-(B+2Q)}{3B}$$

Through further calculation, we find out that $\int_{-\infty }^{\infty }f\left(t\right)dt=1$ is indeed satisfied.

Now, we have enough information to find the average minimum, supported by the calculations in [11]. Up until $P\left(minX_{i}\leq t\right)=1-{\left[1-P\left(X_{i}\leq t\right)\right]}^{n}$ the calculations are the same, but, because of the non-uniformity, $P(X_{i}\leq t)$ is not the same for each *t* in Figure 8. The different values are indicated below.

$$\begin{array}{c} t\epsilon \left[0,Q\right]=>\int_{0}^{t}Adx=At\\ t\epsilon \left[Q,2Q\right]=>AQ+\int_{Q}^{t}2Adx=2At-AQ\\ t\epsilon \left[2Q,B\right]=>3AQ+\int_{2Q}^{t}3Adx=3At-3AQ\\ t\epsilon \left[B,B+Q\right]=>3AB-3AQ+\int_{B}^{t}2Adx=\\ AB-3AQ+2At\\ t\epsilon \left[B+Q,B+2Q\right]=>3AB-AQ+\int_{B+Q}^{t}Adx=\\ 2AB-2AQ+At \end{array}$$

This way, separate density functions are found for each interval, which can be used to calculate E[T] as follows:

$$\begin{array}{c} E\left[T\right]=\int_{0}^{B+2Q}t\times f_{T}\left(t\right)dt=\int_{0}^{Q}t\times f_{T_{a}}\left(t\right)dt+\int_{Q}^{2Q}t\times f_{T_{b}}\left(t\right)dt+\int_{2Q}^{B}t\times f_{T_{c}}\left(t\right)dt+\\ \int_{B}^{B+Q}t\times f_{T_{d}}\left(t\right)dt+\int_{B+Q}^{B+2Q}t\times f_{T_{e}}\left(t\right)dt \end{array}$$

By further calculations, we then obtain that E[T] can be written as:

(5) $$E\left[T\right]=\frac{\left(-B+Q\right){\left(\frac{B-Q}{Q}\right)}^{n}+Q\left(3^{-n}+1\right){\left(\frac{Q}{B}\right)}^{n}-{\left(\frac{-Q+3B}{B}\right)}^{n}\left(-Q+3B\right)3^{-n}+6B}{2n+2}$$

## 6. Experimental Evaluation

This section gives an overview of the measurements that were conducted, preceded by an introduction on the used setup.

### 6.1. Measurement Setup

An initial set of measurements was done using a small-scale setup. The hardware used to implement the BLE Mesh nodes themselves were Nordic nRF52832 modules. Nordic has its own implementation of the Bluetooth Mesh standard [12], which we used to conduct the measurements.

We started with a baseline measurement, using a mesh network of only two nodes. The measurement itself always consists of measuring the communication flow between the these two nodes. This consists of mapping the round trip time for a packet that is sent from the sending node to the receiver node and an acknowledgment that is sent back. Gradually, extra nodes were added, both vertically (to measure the effect of more neighbors in between these two nodes) and horizontally (to measure the effect of more hops between these two nodes). To realize such measurement setups, a specific topology had to be enforced. For this, the Nordic implementation was extended with a filtering mechanism, which enabled filtering on specific addresses at the network layer, resulting in the desired topologies.

**Figure 9:**
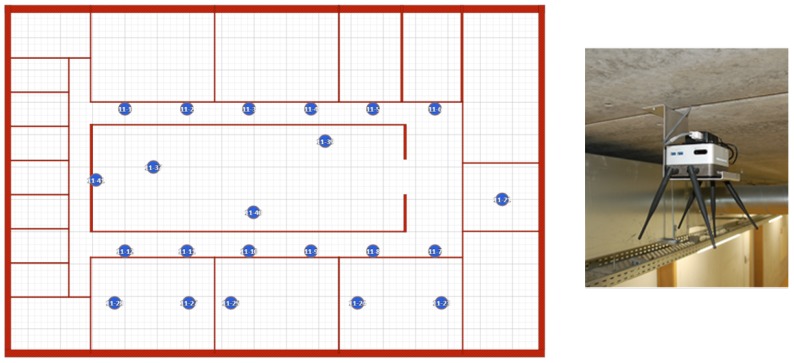
The OfficeLab plan on our floor and a closer look at one of the NUCs.

**Figure 10:**
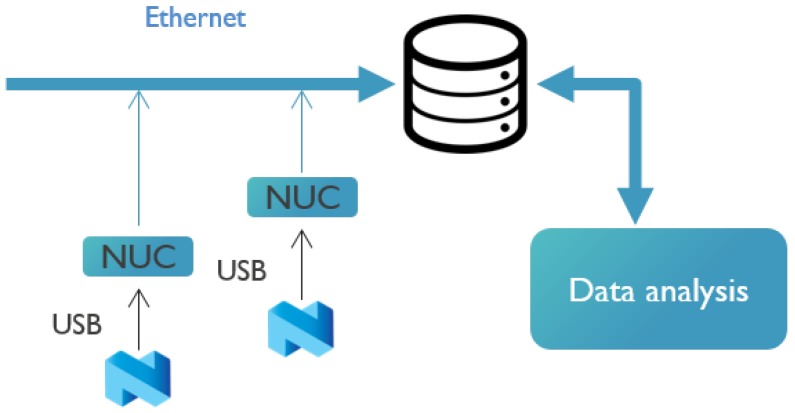
The data collection scheme of the OfficeLab measurement campaign.

Next to this, a second set of measurements was performed in the OfficeLab (OfficeLab Testbed, Ghent University, Ghent, Belgium). This is a testbed that consists of 22 nodes, as shown in Figure 9. The floor size is 40 m × 25 m. Each node corresponds to an Intel NUC, accessible over a wired Ethernet backbone. Each NUC has a nRF52832 module connected to it. Figure 10 indicates how the NUCs capture all USB output intended to evaluate the BLE communication flows. Using a wired Ethernet backbone, all NUCs sent data to a central SQL database. Afterwards, these data were analyzed further, using an in-house visualization tool. The tool exploits the database to visualize the path taken by a specific packet and its corresponding acknowledgment.

Finally, for all measurements, we used the parameters indicated in Table 1.

### 6.2. Baseline Measurement

This measurement served as a baseline for all the following measurements and was done within a minimal mesh network consisting of only two nodes, with one node sending a packet to the other node. Ultimately, this measurement setup gives an average round trip time of 23.653 ms.

Assuming no retransmissions, Equation (2) indicates the three factors influencing the communication, which are the backoff time, processing time and transmit time. To confirm the obtained average RTT with the theoretical analysis, these factors need to examined more closely for this specific setup. Table 1 indicates a maximum backoff of 20 ms, leading to an average backoff time of 10 ms. Concerning the transmit time, the Nordic Bluetooth Mesh stack defines a number of constants regarding the time to enable/disable the radio and switch the channel. Using those constants combined with the packet sizes and throughput speed indicated in Table 1, we obtain 0.490 ms for completing the transmission of the packet on a channel, including starting and stopping the radio, and 0.482 ms for the acknowledgment. This results in a time of 0.490 ms for sending on channel 37, a total time of 2 × 0.490 ms after also sending on channel 38 and a time of 3 × 0.490 ms for completing the transmissions on all three channels. Lastly, there is also some general radio overhead before the first transmission on channel 37, which is another 0.152 ms. As each channel has a 1/3 chance of being used, this results in an average of 2 × 0.490 + 0.152 ms = 1.132 ms for the total transmit time of the packet and 2 × 0.482 + 0.152 ms = 1.116 ms for the total transmit time of the acknowledgment. Finally, by analyzing the different processing parts of the communication, the total processing time for one hop communication is found to be around 1 ms. Ultimately, this give a total theoretical average round trip time of 2 × 10 ms + 1.132 ms + 1.116 ms + 1 ms = 23.248 ms, which is very similar to the obtained experimental result.

### 6.3. Multiple Neighbors Measurement

**Figure 11:**
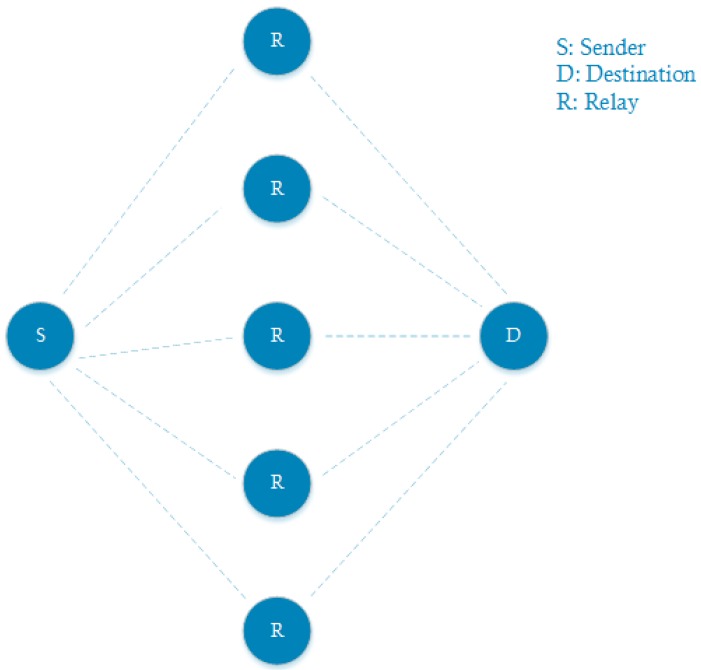
The topology used to conduct the multi-neighbors measurement.

In a realistic mesh network, each node can generally reach more than one other node (or thus neighbors). More neighbors can possibly lead to a lower RTT, because there is a higher probability to have lower backoff times and to receive a packet on channel 37. We verify this through both theoretical and experimental validation, using two-hop communication measurements as indicated in Figure 11, with different amounts of neighbors.

Applied to six neighbors, the theoretical evaluation gives the following. For the backoff time, the sender still counts for 10 ms on average, for sending the packet. Then, the earliest time one of the neighbors can receive and relay the packet is given by 0.152 ms + 0.490 ms for a data packet. Next, according to Equation (5), with Q being equal to 0.490ms and B being equal to 20 ms, the average time after which the neighboring node can start relaying the packet can be calculated, which results in 3.32 ms. Here, we still need to add an additional 0.152 ms + 0.490 ms to account for the preparation of the radio and the actual transmission by that relay. Finally, considering the fact that the destination can listen on any of the three channels, another 0.490 ms needs to be added on average, resulting in a total time of 15,094 ms. Similarly, the total delay for the acknowledgement can be calculated, being 15.07 ms. Finally, considering the fact that the total processing time of sender and receiver for single hop communication is 1 ms and each extra hop adds another 0.8 ms, this results in a total average theoretical RTT of 31.965 ms, opposed to a experimental average of 35.16158 ms.

**Figure 12:**
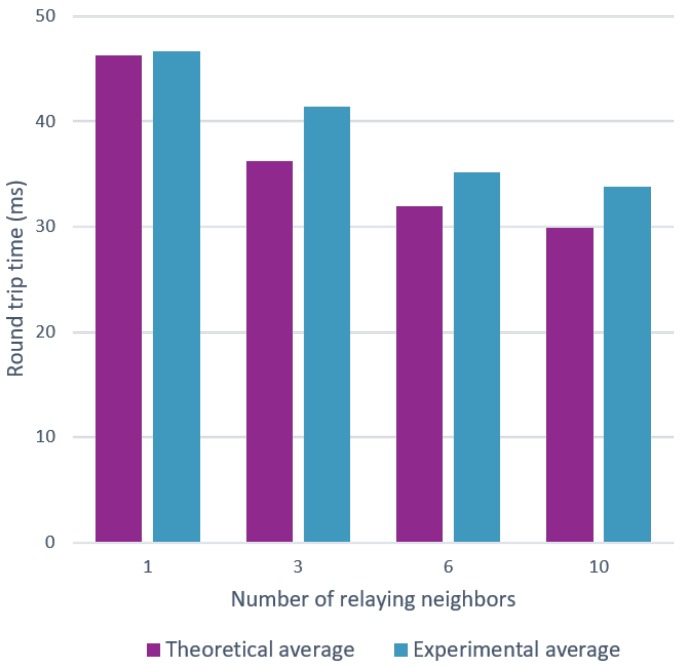
Two hop communication measurements with a varying amount of neighbors.

Figure 12 unites both the theoretical and experimental measurements that were done for several amounts of neighbors. As expected, we observe a positive effect on the round trip time by increasing the amount of relaying neighbors.

### 6.4. Multiple Hops Measurement

**Figure 13:**
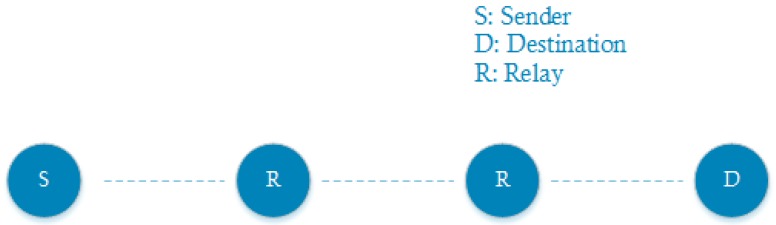
Multi-hop communication with a varying amount of hops.

In larger deployments, it is more realistic for a mesh network to have communication flows that span multiple hops before reaching the intended destination. An increased number of hops also implies a higher round trip time. Again, this effect can be shown via both theoretical and experimental evaluation. An example of one of the forced topologies is indicated in Figure 13.

**Figure 14:**
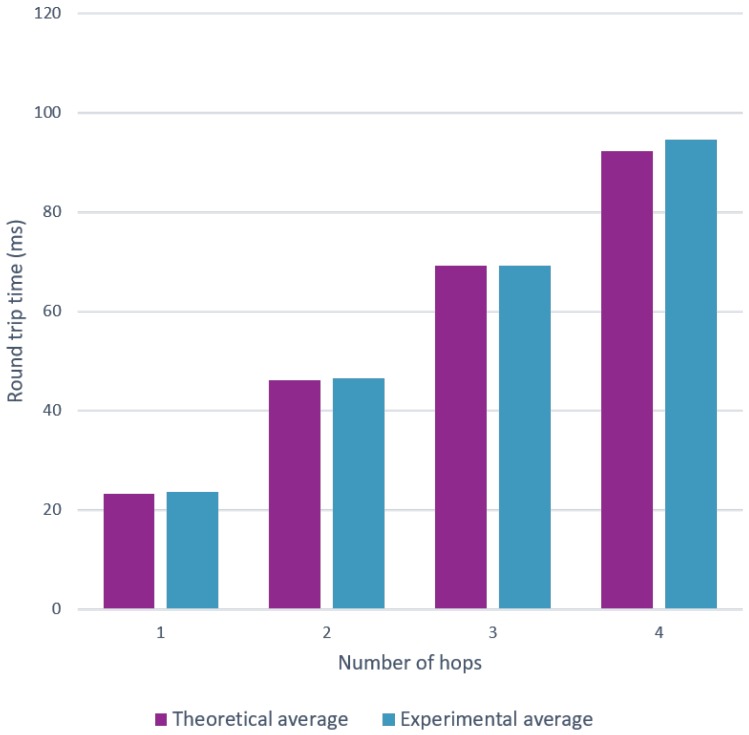
Multi hop communication measurements with a varying amount of hops.

Figure 14 denotes a noticeable effect on the round trip time when the number of hops increases.

**Figure 15:**
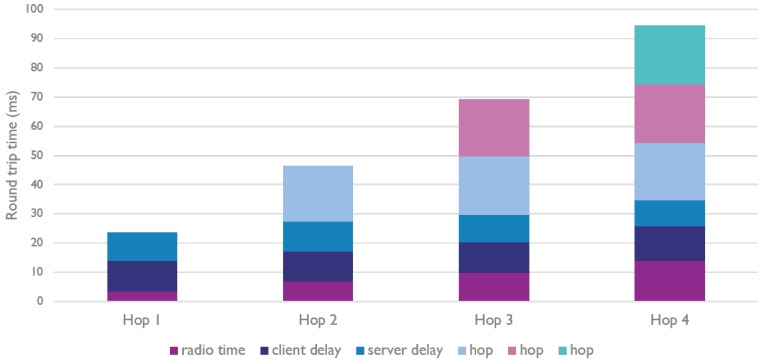
Time distribution for the average round trip time of multi-hop communication in Bluetooth Mesh networks.

When further analyzing the obtained results, we see that there is also a clear pattern in the round trip time for different amount of hops, e.g., the RTT for three hop communication is approximately three times the RTT for single hop communication). This is explained by Figure 15, which shows that the random back off mechanism contributes most to the communication delay. Of course, without this mechanism, the chance to have collisions increases, in turn decreasing the reliability and scalability of Bluetooth Mesh networks.

### 6.5. Retransmission Mechanism

The arbitrary time period before retransmission is application dependent. The Nordic Bluetooth Mesh stack calculates the waiting time via a summation of a baseline period and an extra period that depends on the value of the TTL in the packet. Using the values in Table 1, this gives *200 ms + 4 × 20 ms = 280 ms*. Each time another retransmission is needed for the same packet, the time period is set to the current retransmission time and the variable holding the current retransmission time is doubled. This means that, in our case, 280 ms will be the waiting time for two retransmissions, after which it will always be double the previous waiting time. The standard also defines a timeout period before the packet is considered to be undeliverable, which is 30 s by default. Within this specific timeout period, a total of eight transmissions can be done before the transmission of the packet is abandoned and considered to have failed. This is indicated in Table 2 and was validated through measurements.

### 6.6. Influence of Other BLE Interference

**Figure 16:**
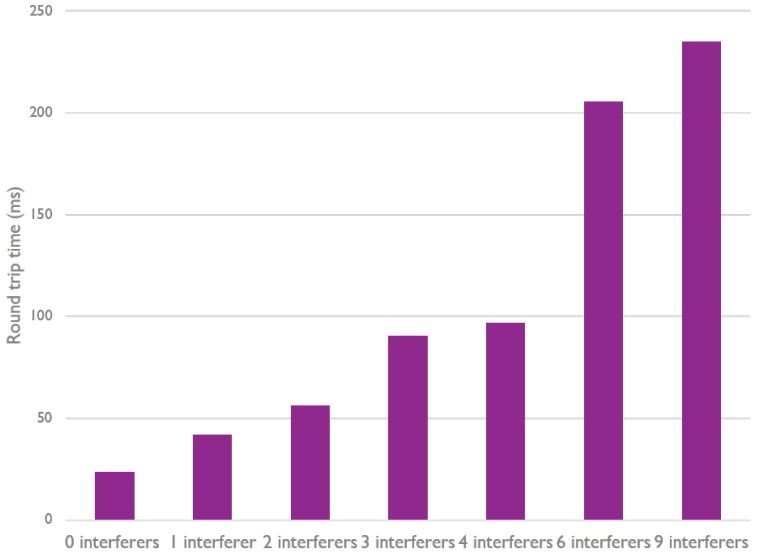
Round trip time of a Bluetooth Mesh network with a varying amount of BLE beacons.

The previous measurements were conducted in a controlled environment. In a more realistic environment (e.g., an office environment), it is possible that wireless communication traffic and other noise from outside the Bluetooth Mesh network has an effect on the communication flows inside the network. Therefore, the following measurement focuses on possible interference from external BLE beacons. Every beacon is configured to broadcast a packet on all three advertisement channels every 20 ms. Again, a small mesh network of only two Bluetooth Mesh nodes that communicate with each other is used. The BLE beacons are placed in range of those nodes and start to broadcast. Figure 16 shows the effect on the RTT, which grows with more interfering BLE beacons.

**Figure 17:**
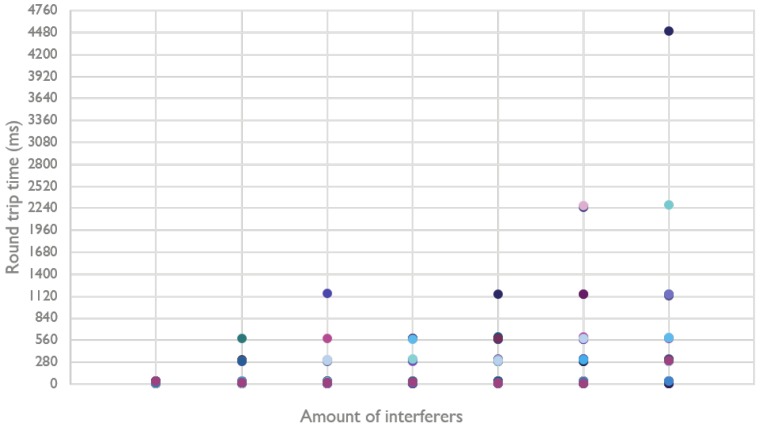
Distribution of the round trip time with a varying amount of BLE beacons.

With nine interfering BLE beacons, the average round trip time is almost 280 ms, which is the first retransmission period for these measurements. As such, it is quite clear that these beacons can disrupt the communication flow, resulting in retransmissions. This is effect is even better illustrated in Figure 17, which shows the distribution of the round trip time for each measurement, per amount of beacons.

By comparing Figure 17 with Table 2, we see that, for only one beacon, there are already some retransmissions needed. This effect increases with the amount of beacons.

### 6.7. Office Lab Measurements

**Figure 18:**
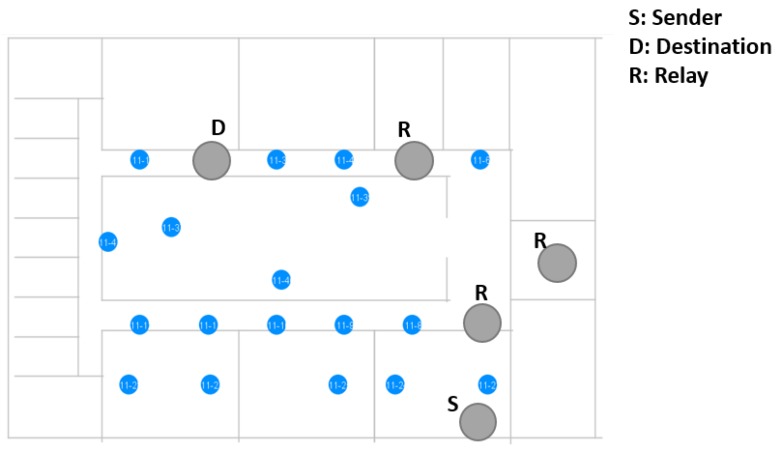
The Bluetooth Mesh network used to conduct experiments.

It is interesting to force a fixed topology and check whether the communication flow behaves as expected. However, it is equally relevant to see how a non-controlled topology behaves in a realistic environment. To demonstrate this, a set of measurements was done in our OfficeLab. Only a part of the testbed was used, as shown in Figure 18.

**Figure 19:**
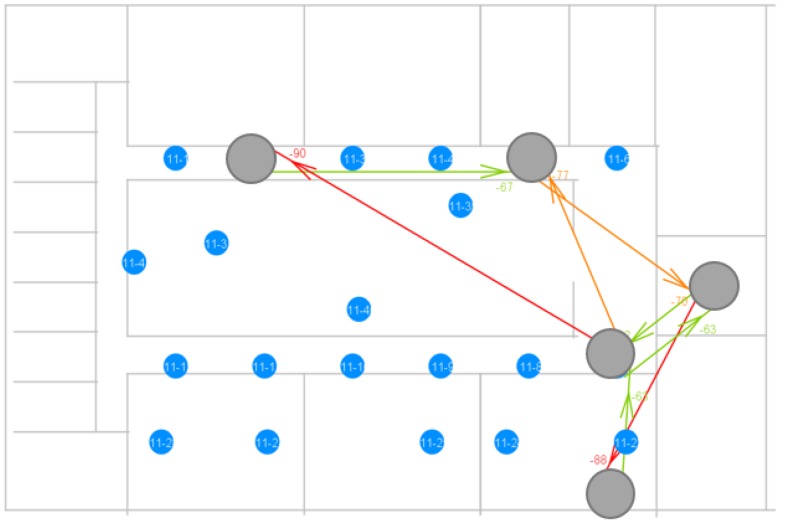
Visualization of a measurement using a physical throughput of 1 Mbps.

Depending on the distance between the nodes in the network, the other traffic going on in the same frequency band and the layout of the building, a specific topology will be generated. Consequently, it is not clear how many hops will be taken when sending a packet from node S to node D and how many hops will be taken to acknowledge that packet from node D to node S at a specific moment in time. As explained, in our testbed, the nodes print timing information over USB when a packet is received (and relayed if possible). Using the setup shown in Figure 9, all USB data were assembled in a SQL database. As such, by analyzing the data in the database, it is possible to reconstruct the path a specific packet and its acknowledgement have taken. Using our in-house visualization tool, the communication path can be shown more clearly. Figure 19 illustrates a measurement done using the parameters given in Table 1.

**Figure 20:**
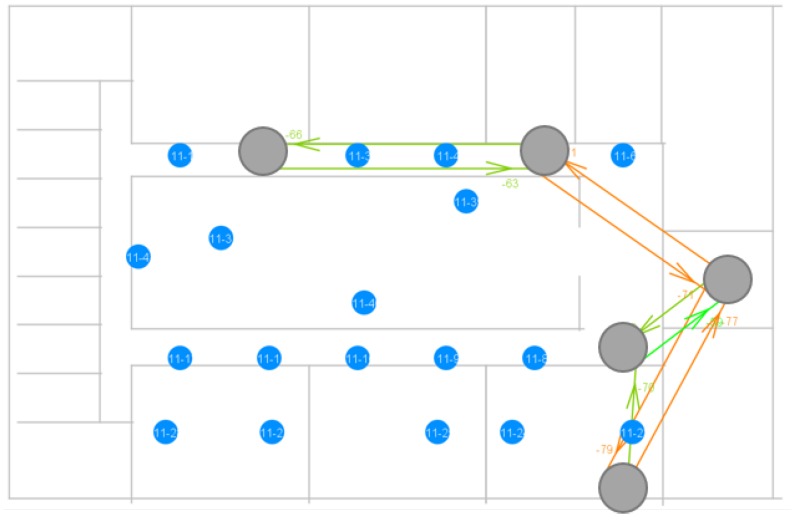
Visualization of a measurement using a physical throughput of 2 Mbps.

The arrows indicate a broadcast transmission and which nodes received it. Their color depends on the RSSI of the received message, with red meaning a weaker signal and green meaning a stronger signal. Nevertheless, in the specific case depicted in the figure above, the sending node was able to reach the destination node in only two hops. The second hop is shown to be a weak signal, but still good enough to reach the destination node and to be correctly interpreted by this node. The Bluetooth Mesh standard uses 1 Mbps physical throughput by default. However, 125/500 kbps (Long Range (LR) feature) or 2 Mbps can also be used, if the right hardware is available. Depending on the use case, higher range or higher throughput could be needed. Figure 20 illustrates what happens to the topology when 2 Mbps is used as the physical throughput.

**Figure 21:**
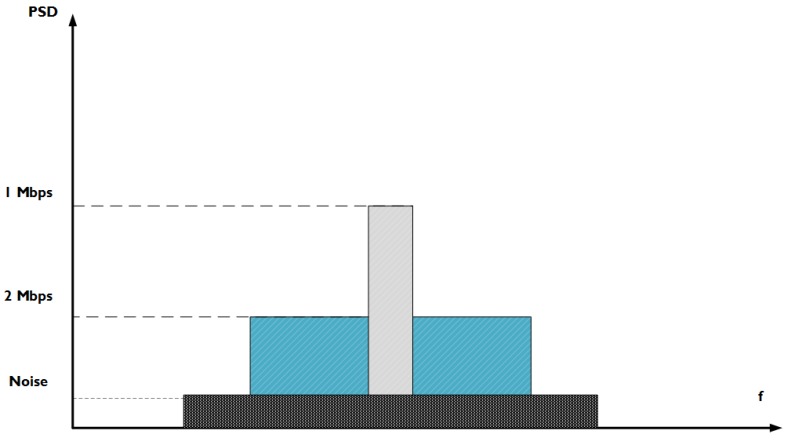
Power density over frequency for 1 Mbps and 2 Mbps.

The network has become less dense, because nodes are no longer able to reach as far as they could when using 1 Mbps. The BLE standard states that the 1 Mbps and 2 Mbps physical throughputs use the same modulation technique, Gaussian Frequency Shift Keying (GFSK), to achieve one bit per symbol. Thus, the symbol rates are, respectively, 1 Msymbol/s and 2 Msymbol/s, to reach the required bit rate. To achieve the higher symbol rate for 2 Mbps, the standard also states that the minimal frequency deviation used with GFSK should be higher. This leads to a higher channel bandwidth being used, out of the 2 MHz channel bandwidth that is available. By plotting the power spectral density (PSD = transmit power / bandwidth used) against the frequency for both symbol rates, as shown by Figure 21, it is clear that 2 Msymbol/s leads to a lower PSD, if the same transmit power is used for both cases (which is the case in our experiments). Because the signal power decreases over distance and the PSD for 2 Msymbol/s is lower, it is clear that the receiver sensitivity for that rate is also lower. This ultimately leads to the smaller coverage range observed. A physical throughput of 2 Mbps can be an option for networks that require a higher throughput in general, but also to make a dense network more sparse.

It could also be interesting to see how the Bluetooth Mesh network reacts to the Long Range (LR) feature, introduced in Bluetooth 5, which uses coding schemes to achieve a much bigger coverage range. Unfortunately, the Bluetooth Mesh stack offered by Nordic does not yet support the LR feature. Intuitively, it can be expected that the communication flow will generally require less hops compared to 1 and 2 Mbps throughput speed, when using the same network.

## 7. Theoretical Model

Finding out the real limits of the Bluetooth mesh standard using experimentation is hard, as it requires dense and/or large network deployments. Therefore, using the knowledge obtained from theory in Section 3 and the experiments in Section 6, we developed a theoretical model for the Bluetooth Mesh standard. The model is inspired by the concept of dynamic graphs [13]. The main difference between a static and dynamic graph, is the fact that the amount of nodes or edges of a graph can change over time. When such a graph is weighted, the node or edge weights can also change over time.

**Figure 22:**
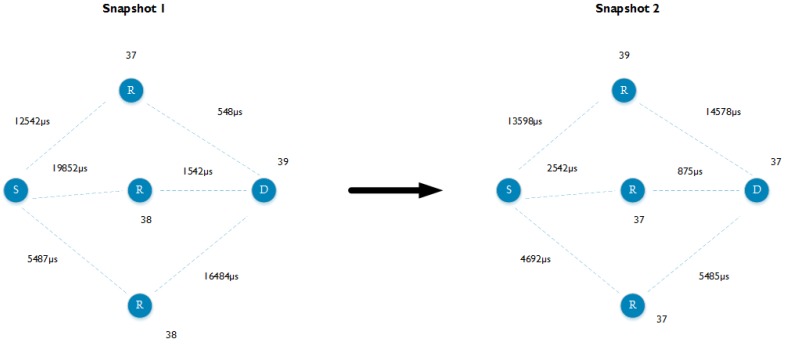
Two snapshots of the mesh network used to conduct the experiments with three neighbors.

The most important factors that influence the communication flow in a Bluetooth Mesh network have been explained in previous sections. Using this knowledge, such a network and its behavior can be simulated using a model. The network is represented by a directed dynamic graph, where snapshots of this graph reflect the dynamics of the network that a communication stream encounters in terms of channel intermediate nodes are listening on and backup value that are used. As such, each node has a weight representing the channel it is currently scanning on and each edge has a weight representing a random backoff time between 0 and the maximum backoff time. Using this snapshot, a shortest path algorithm is used to calculate the end-to-end delay between two nodes and the amount of hops needed. By generating several snapshots, reflecting the randomness in the used channel and backoff values, and calculating the end-to-end delay for each snapshot, an average end-to-end delay for a given mesh network can be calculated. Figure 22 shows two snapshots of the mesh network used to conduct the experiments with three neighbors. In the remainder of this section, we validate the described model using the experiments from Section 6.

**Figure 23:**
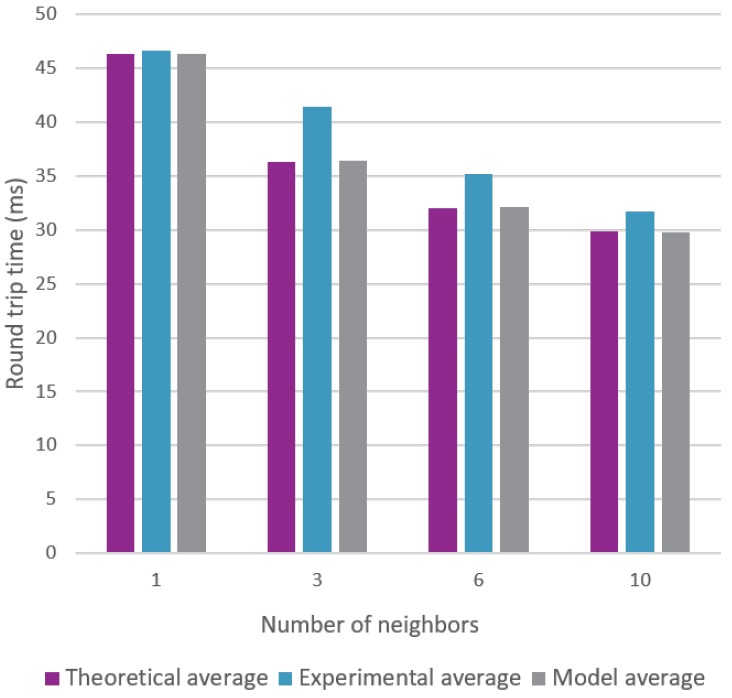
Comparison for the average round trip time using a varying amount of neighbors.

**Figure 24:**
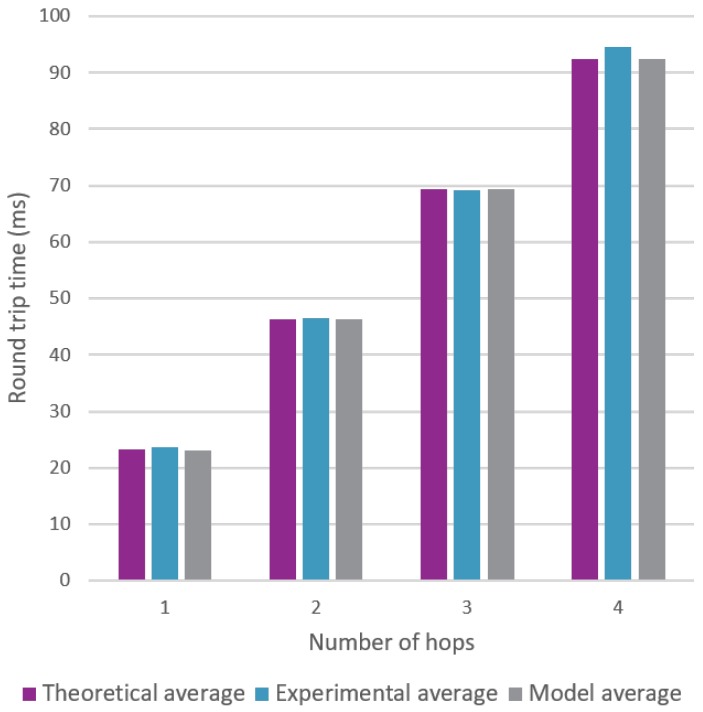
Comparison for the average round trip time using a varying amount of hops.

Both Figure 23 and Figure 24 clearly show that the model is able to replicate the behavior shown by the experiments. However, more importantly, the model is able to produce results that are very close to the theoretical calculations. The average round trip times were found using 5000 snapshots of a topology, which is a lot more than the 100 experiments used in Section 6.

**Figure 25:**
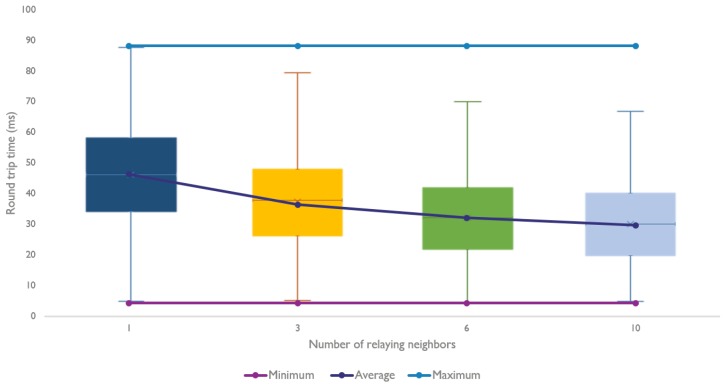
Distribution of the average round trip time for all the snapshots, using a varying amount of neighbors.

**Figure 26:**
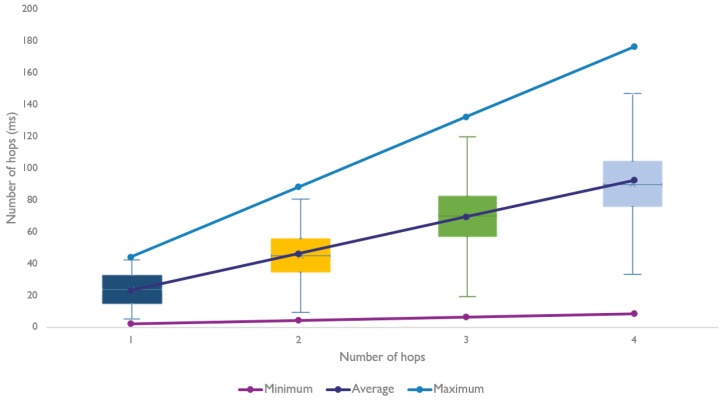
Distribution of the average round trip time for all the snapshots, using a varying amount of hops.

Its also clear what are the maximum and minimum achievable round trip times. The maximum RTT is produced when having the maximum backoff-interval at each backoff time and when each node is receiving on channel 39. The minimum RTT is reached when having 0 ms for each backoff time, with nodes always receiving on channel 37. The RTT given by a snapshot of a certain topology, should always be between this minimum and maximum RTT for that topology. Figure 25 and Figure 26 prove that this is the case for each snapshot used for the validation.

## 8. Conclusions and Future Work

Until recently, the official Bluetooth specification did not have support for mesh capabilities, a property that was present in other competing Internet of Things communication technologies such as Thread or ZigBee. Recently, this gap has been filled in by the Bluetooth Mesh standard, specifying an interoperable solution for BLE-based mesh networking. In this paper, we have given a detailed overview on how this standard operates. It is clear that using Bluetooth Low Energy as the underlying technology for mesh networking has a lot of potential within the Internet of Things. However, the technology still has other issues that need be dealt with. The technology clearly focuses on non-power limited nodes, e.g., smart lighting, to construct the backbone of the mesh network, with the possibility to add power limited nodes to an existing network if needed. The technology in its current state is not suited to implement a mesh network consisting of solely power limited nodes.

Next to this, this paper also evaluated the latency performance of BLE-based meshing using three different methodologies, namely a statistical approach, an experimental assessment and a dynamic graph-based simulation model. The results of the different performance evaluation approaches are on the same line, and can be extended to provide assistance in further studies.

The outcome of our work shows that the backoff mechanism used in the technology, has a lot of impact on the RTT within the network. Increasing the amount of neighbors, i.e., making the network more dense, has a positive effect on the RTT, but a too dense network could also lead to more collisions. Increasing the amount of hops needed, i.e., making the network more sparse has a negative effect on the RTT. Disabling the backoff mechanism decreases this effect but makes the network less scalable and robust. Changing the physical throughput and exposing the network to other technologies, also has a noticeable impact on the performance. As a consequence, it is clear that there are a lot of factors influencing the communication flows within a Bluetooth Mesh network, requiring more advanced management mechanism for optimizing the performance of the mesh network.

Next to this, further studies need to be done regarding other aspects of the technology (security, provisioning, etc.), as well as a performance evaluation of other metrics such as throughput, power consumption, etc. The presented approaches can also be further extended, i.e., by considering intra and inter-technology collisions, in order to asses more larger scale dense networks. We also see opportunities to further look into the use of BLE meshing for more power limited IoT scenarios.

## Figures and Tables

**Table 1:** Simulation Parameters.

Parameter	Value
Transmission power	0 dBm
Scan interval	10 ms
Maximum backoff	20 ms
BLE radio throughput	1 Mbps
Packet size	41 bytes
Acknowledgment size	40 bytes
TTL	4
Base retransmit interval	10 × Maximum backoff
Per TTL extra retransmit interval	Maximum backoff

**Table 2:** All retransmission periods that can occur within a timeout period of 30 s.

(Re) Transmission Number	1	2	3	4	5	6	7	8
**Timeout**	0 ms	280 ms	280 ms	560 ms	1120 ms	2240 ms	4480 ms	8690 ms
